# Cytomegalovirus Infection in Pregnancy

**DOI:** 10.1155/S1064744994000554

**Published:** 1994

**Authors:** Patrick Duff

**Affiliations:** Division of Maternal-Fetal Medicine University of Florida College of Medicine P.O. Box 100294 Gainesville FL 32610-0294 USA

## Abstract

Cytomegalovirus (CMV) infection is of great importance to obstetrician-gynecologists because maternal infection is relatively common and can result in severe injury to the fetus. The greatest risk to the fetus occurs when the mother develops a primary CMV infection in the first trimester. Forty to 50% of infants delivered to mothers with primary CMV infections will have congenital infections. Of these neonates, 5–18% will be overtly symptomatic at birth. Approximately 30% of severely infected infants die, and 80% have severe neurologic morbidity. Eighty-five to 90% of infants will be asymptomatic, and 10–15% of these babies subsequently have sequelae such as visual and auditory defects. If the mother develops a recurrent or reactivated CMV infection during pregnancy, the risk of a severe congenital infection is very low. Perinatal infection, as opposed to congenital infection, may result from exposure to the virus during delivery or lactation and rarely leads to serious sequelae. Antimicrobial therapy and immunotherapy for CMV are, at present, unsatisfactory. Therefore, all patients, pregnant women in particular, must be educated about preventive measures.


Infectious Diseases in Obstetrics and Gynecology 2:146–152 (1994)
© 1994 Wiley-Liss, Inc.

### Cytomegalovirus Infection in Pregnancy

**Patrick Duff**

*Division of Maternal-Fetal Medicine, University of Florida College of Medicine, Gainesville, FL*

ABSTRACT

**Cytomegalovirus (CMV) infection is of great importance to obstetrician-gynecologists because maternal infection is relatively common and can result in severe injury to the fetus. The greatest risk to the fetus occurs when the mother develops a primary CMV infection in the first trimester. Forty to 50% of infants delivered to mothers with primary CMV infections will have congenital infections. Of these neonates, 5–18% will be overtly symptomatic at birth. Approximately 30% of severely infected infants die, and 80% have severe neurologic morbidity. Eighty-five to 90% of infants will be asymptomatic, and 10–15% of these babies subsequently have sequelae such as visual and auditory defects. If the mother develops a recurrent or reactivated CMV infection during pregnancy, the risk of a severe congenital infection is very low. Perinatal infection, as opposed to congenital infection, may result from exposure to the virus during delivery or lactation and rarely leads to serious sequelae. Antimicrobial therapy and immunotherapy for CMV are, at present, unsatisfactory. Therefore, all patients, pregnant women in particular, must be educated about preventive measures.** © 1994 Wiley-Liss, Inc.

KEY WORDS
Epidemiology, perinatal infection, congenital infection, diagnosis, prevention

Cytomegalovirus (CMV) infection is of great importance to obstetrician-gynecologists for several reasons. First, although CMV typically causes either asymptomatic or mildly symptomatic illness in children and adults, it can cause devastating systemic infections in immunocompromised patients, particularly individuals who have received organ transplants or who have human immunodeficiency virus (HIV) infections. Second, the virus can disseminate across the placenta and infect the fetus, even in a woman with a high serum concentration of viral-specific antibody. Third, an assessment of the empiric risk to the fetus can be difficult in any specific patient. Fourth, no consistently effective form of immunotherapy or chemotherapy is available; therefore, physicians must be constantly attuned to the issue of disease prevention.

This article initially reviews the virology and epidemiology of CMV infection. Special emphasis is placed on the accurate classification of maternal, fetal, and neonatal infections. Subsequently, the text considers the prognosis in infected infants and examines several techniques for the prenatal diagnosis of CMV infection. Finally, consideration is given to measures of proven value in reducing the risk of acquiring CMV infection.

### EPIDEMIOLOGY

CMV is a double-stranded DNA virus that replicates within the nucleus of an infected cell. Humans are the only known host for this virus. Like herpes simplex virus, CMV may remain latent in host cells after the initial infection. Recurrent infection is usually due to the reactivation of endogenous latent virus rather than reinfection with a new strain of virus. Cell-mediated immunity is more important than humoral mechanisms in controlling infection.[1]

Address correspondence to Dr. Patrick Duff, Division of Maternal-Fetal Medicine, University of Florida College of Medicine, P.O. Box 100294, Gainesville, FL 32610-0294.

Received March 29, 1994

Accepted June 23, 1994

Review Article

CMV is not highly contagious, and close personal contact is required for infection to occur. *Horizontal transmission* may result from the receipt of an infected organ or blood, from sexual contact, or from contact with contaminated saliva or urine. *Vertical transmission* may occur as a result of transplacental infection, exposure to contaminated genital-tract secretions during delivery, or breast-feeding. The incubation period of the virus ranges from 28 to 60 days, with a mean of 40 days.[1–3]

Among young children, the most important risk factor for infection is close contact with playmates, particularly in the setting of day care. In an early report, Pass and coworkers[4] surveyed 70 children attending a day-care center in Birmingham, AL. Investigators obtained mouth swabs from 29 children and urine specimens from 68. Forty-five percent of the mouth swabs and 53% of the urine cultures were positive for CMV. Nine percent of infants younger than 1 year of age shed the virus in either saliva or urine; 83% of children 1–2 years of age shed the virus.

Jones et al.[5] conducted a similar survey in a regular day-care center and a facility for developmentally delayed children. Twenty-two percent of the children in both centers were viruric, and 11% shed CMV in their saliva. Hutto and coworkers[6] surveyed 47 toddlers attending day care. Fourteen (30%) shed the virus in saliva. The highest rate of salivary excretion (80%) occurred in children 12–24 months of age. Forty percent of the children were viruric. Interestingly, CMV was recovered from 2/70 toy surfaces that were randomly selected for culture. However, the virus was isolated from 5/7 toys immediately after removal from the mouth of a child known to be shedding CMV in saliva. After 10 and 30 min, 4 and 2 toy surfaces, respectively, were still positive.

Infected children clearly pose a risk of transmitting the virus to adult day-care workers. Adler[7] recently examined the risk of seroconversion to CMV among day-care employees. At the time of the initial assessment, 202 workers were seronegative. Within 10 months, 19 (11%) had seroconverted. The risk of seroconversion was greatest among employees who cared for children under 2 years of age.

Small children also pose a risk to members of their own families. Taber et al.[8] performed a serologic study and identified 68 families in which both parents were seronegative for CMV. Over a 3-year period, seroconversion occurred in 1 or more members of 37 families (53%). The mean annual seroconversion rates were approximately 10% for fathers, mothers, and children. The index case was usually a child.

In addition to acquiring infection from young children, adolescents and adults may develop infection as a result of sexual contact. CMV infection is endemic among gay men and heterosexuals with multiple partners.[1,9] Additional risk factors for infection include lower socioeconomic status, history of abnormal cervical cytology, birth outside of North America, first pregnancy at younger than 15 years, and coinfection with other sexually transmitted diseases (STDs) such as trichomoniasis.[10]

### CLINICAL MANIFESTATIONS IN CHILDREN AND ADULTS

Most children who acquire CMV infection are asymptomatic. When clinical manifestations are present, they usually are mild and include malaise, fever, lymphadenopathy, and hepatosplenomegaly. Similarly, most adults with either primary or recurrent CMV infection are asymptomatic. Symptomatic patients typically have findings suggestive of mononucleosis. Respiratory infection is distinctly uncommon in adults with normal immune functions. However, Lipton et al.[11] have reported a case of fatal CMV pneumonitis in an ostensibly healthy postpartum woman. Additional cases of serious CMV infection are likely to occur as a consequence of the increasing prevalence of HIV infection in women.

### DIAGNOSIS OF INFECTION IN ADULTS AND CHILDREN

The diagnosis of CMV infection can be confirmed by the isolation of the virus in tissue culture. The highest concentrations of CMV are usually present in urine and seminal fluid, followed by saliva and breast milk. Several different cell lines have been used to support viral growth, and techniques such as the viral shell assay, immunofluorescent staining, monoclonal antibody, and polymerase chain reaction (PCR) permit the identification of viral antigen within 24 h.[12–15]

Serologic methods also are helpful in establishing the diagnosis of CMV infection provided that the reference laboratory is skilled in performing such tests. In the acute phase of infection, viral-specific IgM antibody is present in serum. IgM titers decline rapidly over a period of 30–60 days. There is no absolute IgG titer that clearly will differentiate acute from recurrent infection. However, a 4-fold or greater change in the IgG titer is consistent with recent acute infection.[1] Other laboratory tests suggestive of CMV infection include a differential white blood cell (WBC) count showing atypical lymphocytes, a low platelet count and elevated serum transaminase concentrations.

### CONGENITAL AND PERINATAL INFECTION

As a result of exposure to either young children or infected sexual partners, approximately 50–80% of adult women in the United States have serologic evidence of past CMV infection. Unfortunately, the presence of antibody is not perfectly protective against vertical transmission; thus, pregnant women with recurrent or primary infection pose special risks to their fetuses.

Fetal and neonatal CMV infections may occur at 3 distinct times: antepartum, intrapartum, and postpartum. Antepartum or congenital infection is the greatest risk to the fetus and is perhaps the most difficult to understand because of the often disparate statistics reported in epidemiologic surveys.

### Congenital (Antepartum) Infection

Congenital CMV infection results from hematogenous dissemination of the virus across the placenta. Dissemination may occur with primary or recurrent (reactived) infection but is much more likely to occur in the former setting. From 1 to 4% of uninfected women seroconvert during pregnancy.[16] In women who acquire primary infection, 40–50% of the fetuses will be infected. Based on work with a guinea-pig model, Kumar and Prokay[17] have concluded that the overall risk of congenital infection is greatest when a maternal infection occurs in the third trimester, but the probability of severe fetal injury is highest when a maternal infection occurs in the first trimester.

Of fetuses with congenital infection, 5–18% will be overtly symptomatic at birth. The most common clinical manifestations and laboratory abnormalities in these infants are listed in Table 1. Approximately 30% of severely infected infants die. Eighty percent of the survivors have severe neurologic morbidity, ocular abnormalities, or sensorineural hearing loss.[18,19] Approximately 85–90% of infants delivered to mothers with primary infection will be asymptomatic at birth. Ten to 15% subsequently develop hearing loss, chorioretinitis, or dental defects within the first 2 years of life. The outcomes of neonates exposed to primary maternal CMV infection are summarized in Figure 1.

Pregnant women who experience recurrent CMV infection are much less likely to transmit infection to their fetuses. Recurrent infection occurs predominantly as a result of reactivation of a latent infection rather than reinfection with a new viral strain. The most recent, and probably clearest, delineation of fetal risk in this situation is the

**TABLE 1. Common clinical findings and laboratory abnormalities in congenital CMV infection[a]**

| Physical findings and laboratory abnormalities | Approximate frequency (%) |
|---|---|
| Physical findings | |
| Enlargement of liver and/or spleen | 52 |
| Petechiae | 51 |
| Intracranial calcifications | 43 |
| Jaundice | 38 |
| Growth restriction | 38 |
| Microcephaly | 27 |
| Chorioretinitis | 15 |
| Sensorineural hearing loss | 15 |
| Mental retardation | 13 |
| Seizures | 10 |
| Laboratory abnormalities | |
| Thrombocytopenia | 52 |
| Hyperbilirubinemia | 35 |
| Increased alanine aminotransferase | 26 |

[a]Information in this table is based upon data presented in references 18 and 19.

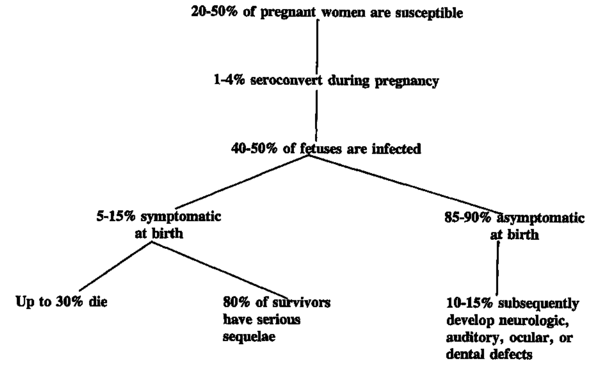

Fig. 1. Frequency and severity of congenital CMV infection following primary maternal infection.

report by Fowler et al.[19] In an excellent epidemiologic investigation, these authors studied 125 women with serologic evidence of primary infection and 64 with recurrent infection. In the former group, 18% of infants were symptomatic at birth. An additional 7% (total 25%) developed at least 1 major sequela within 5 years of follow-up. Two percent died, 15% had sensorineural hearing loss, and 13% had IQs lower than 70. In contrast, none of the infants delivered to mothers with recurrent infection were symptomatic at birth. During the period of surveillance, 8% had at least 1 sequela, but none had multiple defects. The most common sequela was hearing loss. The authors concluded that maternal antibody provided substantial, but not complete, protection against serious fetal infection.

Overall, approximately 40,000 (1%) infants born in the United States each year have congenital CMV infection. Approximately 3,000–4,000 infants are symptomatic at birth, and an additional 4,000–6,000 subsequently have neurologic or developmental problems in the first years of life. CMV infection is now the principal cause of hearing deficits in children. Public-health officials estimate that the annual cost of caring for children with congenital CMV is $1.86 billion.[20]

### Perinatal (Intrapartum and Postpartum) Infection

Perinatal infection may occur *during delivery* as a result of exposure to infected genital-tract secretions. At the time of delivery, up to 10% of pregnant women may be shedding CMV in cervical secretions and/or urine. Twenty to 60% of exposed fetuses may subsequently shed CMV in their pharynx and/or urine. The incubation period for this form of infection ranges from 7 to 12 weeks, with an average of 8 weeks. Fortunately, infected infants rarely have serious sequelae from infection acquired during delivery.[18,21]

Perinatal infection also may develop as a *result of breast-feeding*. Stagno et al.[22] surveyed 278 women who had recently delivered and who agreed to provide samples of breast milk. Thirty-eight (13%) had CMV isolated at least once from colostrum or milk. Twenty-eight of these women were shedding CMV only in breast milk. Nineteen of their neonates were breast-fed, and 11 (58%) acquired CMV infection despite the presence of neutralizing antibody in breast milk. Fortunately, serious sequelae did not occur in infected infants.

### DIAGNOSIS OF CONGENITAL INFECTION

Several techniques are now available to evaluate the fetus with suspected congenital CMV infection. Katz et al.[23] were the first to report the association between elevated second-trimester maternal serum alpha-fetoprotein (MSAFP) and congenital infection. They were not able to define the precise explanation for elevation in MSAFP. In view of the nonspecific nature of this test and the multiple other causes for elevated concentrations of this protein, MSAFP screening should not be considered a useful test for the diagnosis of congenital CMV infection.

Much attention in recent years has focused on the analysis of amniotic fluid (AF) and fetal serum as a means to diagnose congenital infection. Several authors have compared the relative value of the following diagnostic tests: viral culture of AF and fetal serum, determination of total IgM concentration in fetal serum, identification of anti-CMV IgM in fetal serum, and assessment of fetal liver-function tests. These reports uniformly have supported the superiority of AF culture in confirming the diagnosis of congenital CMV infection.

Lange and coworkers[24] were the first to report the successful diagnosis of congenital infection by assessment of fetal blood. They performed cordocentesis on a hydropic fetus at 25 week gestation. The fetal blood smear showed severe erythroblastosis. Total IgM concentration was normal, but viral-specific IgM antibody to CMV was detected by radioimmunoassay.

Hohlfeld et al.[12] subsequently described their assessment of 15 women with documented primary CMV infection in pregnancy. Eight fetuses were infected. All were correctly identified by the detection of antigen in AF by the shell-viral assay. In each instance, the subsequent viral culture was positive. Only 4 fetuses (50%) had increased total IgM concentrations and abnormal liver-function tests. Two fetuses had thrombocytopenia, and none had positive viral blood cultures. The finding of a negative AF culture was 100% specific in predicting the absence of congenital infection.

Lynch and coauthors[14] recently described their experience with the assessment of 12 patients, 7 of whom had serologically confirmed primary infection and 5 of whom were evaluated because of abnormal sonographic findings. Eleven of the patients had amniocenteses and cordocenteses. Of the 7 women with primary CMV infection, only 1 had a confirmed fetal infection. This fetus had a normal hematocrit and platelet count, negative IgM-specific antibody, an elevated gamma-glutamyltranspeptidase (GGTP) concentration, and a positive AF culture. In the group of 5 women with abnormal sonograms, all of the fetuses were infected. Four fetuses (80%) had positive AF cultures, and none had a positive blood culture. One had thrombocytopenia, 3 had elevated GGTP concentrations, and 4 had elevated total IgMs.

Lamy et al.[13] reported a large survey of 861 pregnant women who initially were seronegative for CMV. Seroconversion occurred in 20 (2.3%) during pregnancy. Seven of these women agreed to have invasive diagnostic testing. Five of 7 AF cultures were positive; only 3 fetuses had IgM-specific antibody.

In the most recent and largest series of invasive diagnostic testing for congenital CMV infection, Donner et al.[15] assessed 52 fetuses at risk for congenital CMV infection. Sixteen fetuses were infected, 13 of whom were diagnosed antenatally. Thirteen of 16 (81%) had at least 1 abnormal test. The detection of virus in AF by culture or PCR methodology correctly identified 12 (75%) infected fetuses. Four patients required 2 amniocenteses to establish the diagnosis. Nine of 16 (56%) fetuses had viral-specific IgM antibody in cord blood. Six of 11 (55%) fetuses who had hematologic assays were thrombocytopenic. A negative AF culture was 100% specific in identifying an unifected fetus.

Although identification of CMV in AF appears to be the most sensitive and specific test for diagnosing congenital infection, it does not necessarily identify the *severity* of fetal injury. This issue is obviously of great importance in counseling parents about the prognosis for their infant. Fortunately, detailed sonography can be invaluable in providing information about the severity of fetal impairment. The principal sonographic findings suggestive of serious fetal injury include microcephaly, ventriculomegaly, intracerebral calcifications, hydrops, growth restriction, and oligohydramnios.[25] In addition, unusual findings that may also indicate a severely infected infant include fetal heart block,[26,27] intraabdominal echodensities,[28] meconium peritonitis,[29] and isolated serous effusions.[25] Clinicians should be aware that the ultrasound examination may be normal early in the course of fetal infection. Therefore, fetuses at risk should have repeat examinations to determine if anomalies are apparent.

### TREATMENT AND PREVENTION

At the present time, a vaccine for CMV is not available. Antiviral agents such as ganciclovir and foscarnet have moderate activity against CMV, but their use is limited primarily to the treatment of severe infections in immunocompromised patients. Accordingly, obstetrician-gynecologists should focus most of their attention on educating patients about preventive measures.

One of the most important interventions is helping patients understand that CMV infection can be an STD and that sexual promiscuity significantly increases an individual's risk of acquiring the infection. Individuals who have multiple sexual partners should be counseled that latex condoms provide an effective barrier to the transmission of CMV.[29,30] Another important intervention is educating health-care workers, day-care workers, elementary-school teachers, and mothers of young children about the importance of simple infection control measures such as handwashing and proper cleansing of environmental surfaces. Obstetricians and pediatricians must be consistently aware of the importance of transfusing only CMV-free blood products to fetuses, neonates, pregnant women, and immunocompromised patients and of screening potential donors of organs and semen for CMV infection.[2] Finally, health-care workers must adhere to the principles of universal precautions when treating patients and handling potentially infected body fluids.[16]

At the present time, there are several reasons why routine prenatal screening for CMV infection is not recommended. First, laboratory resources may become overwhelmed if all pregnant women are screened. Second, if laboratories do not insure a high level of quality control, the interpretation of serologic tests may be confusing and may lead to incorrect, and irreversible, interventions such as pregnancy termination. When evaluating test results, clinicians must be aware that IgM-specific antibody may persist for up to 60 days after the acute infection. Therefore, they must carefully assess the patient's clinical history to determine the most likely time of infection with respect to conception. Third, neither antiviral chemotherapy nor immunoprophylaxis is available to protect the fetus or neonate. Accordingly, screening should be limited to women who have symptoms suggestive of acute CMV infection, who have had definite occupational exposure to CMV, or who are immunocompromised.

### CONCLUSIONS

CMV infection is the most common congenital infection in the United States, affecting approximately 1% of all neonates. The most serious fetal injuries result from primary maternal infection. Mothers with recurrent infections are much less likely to transmit infection to their fetuses. The most valuable tests for confirming the diagnosis of congenital CMV infection and determining the severity of fetal injury are culture of AF and ultrasonography. Infants also may acquire CMV infection as a result of exposure to CMV during delivery or during breast-feeding. Serious sequelae from this type of infection are much less common than following primary infection. Antimicrobial therapy and immunotherapy for CMV infection are, at present, unsatisfactory. Therefore, patients must be educated to avoid high-risk behavior.

### REFERENCES

1. Betts RF: Cytomegalovirus infection epidemiology and biology in adults. Semin Perinat 7:22–30, 1983.
2. Wilhelm JA, Malter L, Schopfer K: The risk of transmitting cytomegalovirus to patients receiving blood transfusions. J Infect Dis 154:169–171, 1986.
3. Stagno S, Pass RF, Dworsky ME, Alford CA: Congenital and perinatal cytomegalovirus infections. Semin Perinat 7:31–42, 1983.
4. Pass RF, August AM, Dworsky M, Reynolds DW: Cytomegalovirus infection in a day care center. N Engl J Med 307:477–479, 1982.
5. Jones LA, Duke-Duncan PM, Yeager AS: Cytomegaloviral infections in infant-toddler centers: Centers for the developmentally delayed versus regular day care. J Infect Dis 151:953–955, 1985.
6. Hutto C, Little EA, Ricks R, Lee JD, Pass RF: Isolation of cytomegalovirus from toys and hands in a day care center. J Infect Dis 154:527–530, 1986.
7. Adler SP: Cytomegalovirus and child day care. N Engl J Med 321:1290–1296, 1989.
8. Taber LH, Frank AL, Yow MD, Bagley A: Acquisition of cytomegaloviral infections in families with young children: A serological study. J Infect Dis 151:948–952, 1985.
9. Demmler GJ, Schydlower M, Lampe RM: Texas, teenagers, and CMV. J Infect Dis 152:1350, 1985.
10. Chandler SH, Alexander ER, Holmes KK: Epidemiology of cytomegaloviral infection in a heterogeneous population of pregnant women. J Infect Dis 152:249–256, 1985.
11. Lipton SD, Bryant J, Saed F, Fontillas G: Fatal case of cytomegalovirus pneumonitis in a postpartum woman. Obstet Gynecol 57:670–673, 1981.
12. Hohlfeld P, Vial Y, Maillard-Brignon C, Vaudaux B, Fawer CL: Cytomegalovirus fetal infection: Prenatal diagnosis. Obstet Gynecol 78:615–618, 1991.
13. Lamy ME, Mulongo KN, Gadisseaux JF, Lyon G, Gaudy V, VanLierde M: Prenatal diagnosis of fetal cytomegalovirus infection. Am J Obstet Gynecol 166:91–94, 1992.
14. Lynch L, Daffos F, Emanuel D, et al.: Prenatal diagnosis of fetal cytomegalovirus infection. Am J Obstet Gynecol 165:714–718, 1991.
15. Donner C, Liesnard C, Content J, Busire A, Aderca J, Rodesch F: Prenatal diagnosis of 52 pregnancies at risk for congenital cytomegalovirus infection. Obstet Gynecol 82:481–486, 1993.
16. Adler SP: Cytomegalovirus and pregnancy. Curr Opin Obstet Gynecol 4:670–675, 1992.
17. Kumar ML, Prokay SL: Experimental primary cytomegalovirus infection in pregnancy: Timing and fetal outcome. Am J Obstet Gynecol 145:56–60, 1983.
18. Stagno S, Pass RF, Dworsky ME, et al.: Congenital cytomegalovirus infection. N Engl J Med 306:945–949, 1982.
19. Fowler KB, Stagno S, Pass RF, Britt WJ, Boll TJ, Alford CA: The outcome of congenital cytomegalovirus infection in relation to maternal antibody status. N Engl J Med 326:663–667, 1992.
20. Dobbins JG, Stewart JA, Demmler GJ: Surveillance of congenital cytomegalovirus disease, 1990–1991. MMWR 41:35–44, 1992.
21. Reynolds DW, Stagno S, Hosty TS, Tiller M, Alford CA: Maternal cytomegalovirus excretion and perinatal infection. N Engl J Med 289:1–5, 1973.
22. Stagno S, Reynolds DW, Huang ES, Thames SD, Smith RJ, Alford CA: Congenital cytomegalovirus infection. N Engl J Med 296:1254–1258, 1977.
23. Katz V, Cefalo RC, McCune BK, Moss MK: Elevated second trimester maternal serum alpha-fetoprotein and cytomegalovirus infection. Obstet Gynecol 68:580–581, 1986.
24. Lange I, Rodeck CM, Morgan-Capner P, Simmons A: Prenatal serological diagnosis of intrauterine cytomegalovirus infection. Br Med J 284:1673, 1982.
25. Grose C, Weiner CP: Prenatal diagnosis of congenital cytomegalovirus infection: Two decades later. Am J Obstet Gynecol 163:447–450, 1990.
26. Lewis PE, Cefalo RC, Zaritsky AL: Fetal heart block caused by cytomegalovirus. Am J Obstet Gynecol 136: 967–968, 1980.
27. Karn K, Julian TM, Ogburn PL: Fetal heart block associated with congenital cytomegalovirus infection. J Reprod Med 29:278–280, 1984.

28. Forouzan I: Fetal abdominal echogenic mass: An early sign of intrauterine cytomegalovirus infection. Obstet Gynecol 80:535–537, 1992.
29. Pletcher BA, Williams MK, Mulivor RA, Barth D, Linder C, Rawlinson K: Intrauterine cytomegalovirus infection presenting as fetal meconium peritonitis. Obstet Gynecol 78:903–904, 1991.
30. Katznelson S, Drew WL, Mintz L: Efficacy of the condom as a barrier to the transmission of cytomegalovirus. J Infect Dis 150:155–157, 1984.
